# My Heart and CKD: Pathway to Implementing a Decision Aid for Patients With CKD and Coronary Artery Disease

**DOI:** 10.1177/20543581251389602

**Published:** 2026-02-28

**Authors:** Julie N. Babione, Pantea Javaheri, Denise Kruger, Todd Wilson, Winnie Pearson, Maureena Loth, Violet March, Wayne Gerber, Bryan J. Har, Michelle M. Graham, Stephen B. Wilton, Krystina B. Lewis, Matthew T. James

**Affiliations:** 1 Department of Medicine, Cumming School of Medicine, University of Calgary, AB, Canada; 2 Department of Community Health Sciences, Cumming School of Medicine, University of Calgary, AB, Canada; 3 Department of Cardiac Sciences, Cumming School of Medicine, University of Calgary, AB, Canada; 4 Libin Cardiovascular Institute, Cumming School of Medicine, University of Calgary, AB, Canada; 5 Department of Medicine, Faculty of Medicine and Dentistry, University of Alberta, Edmonton, Canada; 6 Mazankowski Heart Institute, Faculty of Medicine and Dentistry, University of Alberta, Edmonton, Canada; 7 O’Brien Institute for Public Health, Cumming School of Medicine, University of Calgary, AB, Canada; 8 School of Nursing, University of Ottawa, ON, Canada; 9 University of Ottawa Heart Institute, University of Ottawa, ON, Canada

**Keywords:** shared decision-making, decision aid, chronic kidney disease, coronary artery disease

## Abstract

**Background:**

Patients with chronic kidney disease (CKD) at times must decide whether to take an invasive approach to management of coronary artery disease (CAD), which involves procedures such as angiography, angioplasty, and surgery, versus attempt management with medications alone. ***My Heart and CKD*** is a decision aid to support shared decision-making (SDM) between patients and their care providers in these situations. This report describes the pathway to implement ***My Heart and CKD***, and key learnings that have emerged through this process.

**Methods:**

Human-centered design was used to develop the decision aid while concurrently identifying design features that could facilitate its incorporation within patient-physician clinical encounters. Interviews exploring use of the decision aid with patients and care providers were qualitatively analyzed according to the theoretical domains framework to identify barriers and facilitators to implementation. Simulated encounters between patient and physicians were used for pre-clinical testing and to identify additional training and resources that could support effective implementation.

**Key Findings:**

Implementation insights overlapped with decision-aid design input and influenced key design elements of ***My Heart and CKD*** as well as the development of implementation strategies to facilitate its clinical use. An overarching theme that influenced development and implementation was the concept that the decision aid be designed for use by patients and physicians together, to support the bidirectional communication and relationship building intended with SDM. This influenced design features to support varying use preferences and contexts, encompassing both digital and paper-based forms for use. Implementation considerations also influenced order and arrangement of content in ***My Heart and CKD*** to enhance integration of all stages of SDM into varying clinical environments. Additional training and implementation resources, including an accredited continuous medical education program for care providers, were identified as additional facilitators necessary to support implementation.

**Limitations:**

Clinical evaluation has not yet been completed.

**Implications:**

***My Heart and CKD*** is intended to support personalized, patient-centered care by providing patients with CKD and CAD and their care providers with tools to engage in SDM. Further research will evaluate the effectiveness of its implementation and impact on the quality of decision-making.

## Background

Coronary artery disease (CAD) commonly accompanies chronic kidney disease (CKD), and can result in hospitalizations, reduced quality of life, shortened survival, and has implications for receiving a kidney transplant.^1^ Patients with CKD and CAD often must decide whether to take an invasive approach to management, which involves procedures such as angiography, and revascularization procedures to diagnose and treat heart disease, versus attempting optimal medication management alone. This can be a challenging decision for patients and care providers to make, due to the complex trade-offs in the benefits of these procedures for treating heart disease versus their unique risks to the kidney health of people with CKD.^2^

In earlier phases of work, our team engaged with patients with CKD and CAD, and their care providers to develop patient-oriented solutions to address this issue. As a project funded by the patient-oriented research network, Canadians Seeking Solutions for Chronic Kidney Disease (Can-SOLVE CKD), patient partners with lived experience guided all aspects of our research. We first conducted qualitative studies with patients and health care providers to characterize their experiences with CKD and CAD and identify potential strategies to overcome identified challenges in care.^2^ We then developed and validated predictive models to provide patients and their care providers with personalized benefit-risk information under different treatment options for CAD. We also conducted a discrete choice experiment to better understand the values patients placed on the attributes of different treatment options, and how patient preferences varied.^3^ Finally, we used human-centered design (HCD),^4-8^ informed by International Patient Decision Aid Society^9,10^ and the Ottawa Decision Support Framework,^11^ to synthesize current knowledge within a decision aid that we have named ***My Heart and CKD***. This web-based decision aid, available at www.myheartandckd.ca, is intended to support shared decision-making (SDM)^12-14^ between patients with CKD and CAD and their care providers, within a context of trust-building that empowers patients in decision-making by providing personalized information and promoting respect for patients’ values and preferences.

This initiative has now progressed to implementation and evaluation of a SDM approach to invasive versus medical CAD management decisions, supported by the ***My Heart and CKD*** decision aid. Knowledge mobilization and implementation have been prominent throughout our work. Important insights from patients and care providers originated during the design and development of the decision aid and have carried through to current pilot testing the impact of the tool on patient and care provider experiences. In this paper, we describe the pathway taken to implement ***My Heart and CKD*** into clinical care in Canada, and key learnings that have emerged through our work to date.

## Methods

This project’s activities revolve around Can-SOLVE CKD’s 4 pillars of: (1) implementation science/knowledge mobilization, (2) Indigenous cultural competency, (3) incorporation of equity, diversity, and inclusion (EDI) principles in knowledge mobilization and implementation efforts, and (4) patient engagement and capacity building. The ways each of these 4 pillars have been applied are described here.

### Pillar 1: Implementation Science/Knowledge Mobilization

To ensure capacity within the project team to support implementation, 3 team members completed courses on “The How of Creating Sustainable Change” and “Implementation Scale and Spread” offered by the Centre for Implementation (https://thecenterforimplementation.com/). This allowed us to capture early insights into barriers and facilitators to implementing ***My Heart and CKD***, brought forward by patients and care providers starting from its development. During the design and development phase, we created and refined successive iterations of the decision aid over 3 phases that included interviews with patients and care providers, with review of each iteration. As this process progressed and the decision aid took shape, the focus of discussions increasingly shifted from the design of the tool itself to how the decision aid would be implemented into clinical practice and within workflows in outpatient clinics and hospital settings. Noting these emerging areas of discussion during the development process, we purposefully analyzed the data from these interviews to identify themes related to implementation. Transcripts from all phases were analyzed using content analysis, with implementation-related content deductively categorized using the theoretical domains framework (TDF).^15,16^ Suggestions to facilitate implementation approaches were integrated into the decision aid as it evolved and these findings were also used to identify additional implementation supports that would be needed by patients and physicians to effectively use the decision aid, including a collection of continuous medical education (CME)-accredited training materials and implementation guidance resources for users.

To further augment our implementation strategy, we conducted simulated patient-physician encounters for pre-clinical testing of the decision aid. We created 4 unique patient personas and used them with 2 patients and 2 physicians to simulate and observe encounters using the decision aid in hypothetical outpatient clinic and hospital settings. The sessions were observed by the research team, and the patients and physicians who participated in the simulated clinical encounters were debriefed to understand their experience incorporating the decision aid into a patient-physician encounter, barriers to use during the encounter, and strategies to overcome challenges with its integration. Input from this activity was used to make final refinements to the decision aid and identify additional accompanying implementation training materials and guidance resources required to support clinical use.

We are now conducting an implementation pilot trial that will introduce and evaluate the ***My Heart and CKD*** decision aid in 10 clinical cardiology practices in Canada. In this trial, we will evaluate implementation success, including the feasibility, acceptability, and satisfaction with use of the decision aid from the perspective of patients and health care providers, in addition to the effects of the decision aid on the quality of SDM and decision-making confidence within real world care.

### Pillar 2: Indigenous Cultural Competency

We have respectfully worked with Indigenous peoples and engaged representatives of Indigenous communities in the Treaty 7 territories of the Tsuut’ina First Nation, and the Stoney Nakoda First Nation to learn how we can promote SDM using the ***My Heart and CKD*** decision aid in a culturally safe and relevant manner. Training and education of our team through courses from Indigenous Canada, Ownership Access and Privacy (OCAP) training, and resources developed and curated by the Can-SOLVE CKD network (https://cansolveckd.ca/our-work/indigenous-initiatives/) provided our team with a knowledge base from which to apply our work in this area. With support from the Can-SOLVE CKD Network Indigenous Peoples Engagement in Research Circle (IPERC) and Indigenous Liaison Manager, we were introduced and partnered with an Indigenous Knowledge Keeper and an Indigenous person with lived experience with CKD to explore the relevance of the ***My Heart and CKD*** decision aid to Indigenous people experiencing CKD and CAD.

Through in-person meetings, we introduced the objective of our project to support SDM for people with CKD and CAD. Together, we discussed personal and historical experiences of Indigenous peoples with health care, identifying racism, bias and injustices, and barriers to access to care as contributors to distrust of health care providers and the health system by Indigenous peoples. Using the ***My Heart and CKD*** decision aid and promoting SDM was viewed as an approach that could promote a space for trust-building between patients and their health care providers, and more patient-centered care. We identified that introducing SDM and implementation of the decision aid provides an opportunity to embed important teaching on cultural safety and the “6 Rs” of Respect, Relevance, Reciprocity, Relationships, Reflection, and Reconciliation (www.cansolveckd.ca) within our training and implementation program for health care providers. These teachings were integrated into our CME-accredited education program for health care providers to accompany implementation of ***My Heart and CKD***. Adjustments made to the education program included: (1) addition of patient partner insights on relevance of Indigenous cultural competency and safety within our CME program and (2) inclusion of Indigenous ways of knowing and the importance of the 6 Rs within our health care provider training materials.

### Pillar 3: Incorporating EDI Principles and Health Equity

Finding optimal treatment approaches to cardiovascular disease and heart health was previously identified as a top 10 research priority by patients with CKD and health care providers in Canada.^17^ We have approached this topic using HCD^4,5,18,19^ and SDM^13,14,20^ frameworks that promote respect for each person’s unique experiences, values, and preferences for their care. These frameworks are well suited to support understanding personal and systemic contexts and to deliver equitable care and outcomes for all patients. Building upon these objectives, our project naturally progresses to embed EDI-promoting processes within implementation practices. Existing evidence demonstrates that decision aids can improve patient knowledge, reduce their decision uncertainty, improve patient experience, and value-congruent treatment decisions.^21-23^ At the same time, we recognize that thoughtful implementation is also needed, so that decision aids do not exacerbate existing inequities and biases in care delivery.

We developed the ***My Heart and CKD*** website to interface with Google Translate to maximize its accessibility to users with a diversity of spoken languages. Paper-based resources for patients participating in the implementation trial were also translated into French. We identified sex and gender disparities in cardiovascular care as particularly relevant to our initiative because women frequently experience heart disease differently, and there is underrepresentation of females in past studies. Furthermore, women appear to approach health decision-making differently than men, which may be due to sociocultural factors. Research has suggested women receive much less information than they want from health professionals and a lower quality of decision support has been described being provided to females.^24^ Gender differences have also been reported in how physicians access and process information, with males relying more on heuristics, and women allocating more time to evaluating information.^25^ Recognizing these gender disparities, we established a sex and gender-balanced research team in composition of patient partners, clinicians, and researchers, and sought to achieve balanced representation of sexes of patients and care providers among study participants. In our implementation pilot trial, we have pre-specified sex and gender-based analyses to explore differences in the effect of the decision aid on outcome measures of both patient and physician experiences.^26^ Sex and gender differences will be explored, including those of patient-physician sex and gender concordance,^27^ for patient participant evaluations of decision-making quality and decisional conflict.

### Pillar 4: Patient Engagement and Capacity Building

Patient partners with lived experience with CKD and CAD meaningfully contributed to the project from its outset by providing valuable input on the development of the decision aid, co-designing its implementation and evaluation plan, and in disseminating knowledge about the project to study participants and the public. Strategies used to engage patient partners included recurring full team meetings focusing on co-design and creation, participation in development of the education and implementation support resources, patient consent forms, and development of educational videos. A noteworthy contribution of patient partners has been their involvement in the development of the project evaluation framework. This involved meetings to prioritize the primary and secondary outcomes for the implementation pilot trial, and selection of the instruments used to measure patient experience, quality of SDM, and decision conflict from the patient perspective. One patient partner described the development and implementation planning meetings to the Can-SOLVE CKD Research Operations and Knowledge Translation (ROCKeT) Committee as follows:

The learning process of the decision tool’s purpose was interesting: learning more about the interventions for heart disease and the relation to renal function. As a patient partner, I helped to review and reword materials and asked questions that caused the research team to “rework” a part. I don’t feel there were any challenges for me, the team was welcoming and ensured patient partners understood the importance of testing out the tool, and very open to any feedback. Team meetings were well attended, I thought, and it was nice to see everyone coming together for this project, whether you were a researcher, doctor, patient or nurse.**—Patient Partner**

## Key Findings

Thirty-two patients and 18 physicians provided implementation perspectives for the decision aid. Among patient participants, 47% were less than 65 years of age and 47% were women. Among the physician participants, 72% were less than 50 years of age and 22% were women. Supporting quotes and themes related to implementation planning across the domains of capability, motivation, and opportunity, organized within the TDF,^16^ are shown in Table 1.

**Table 1.** Insights From Patients and Clinicians According to the Theoretical Domains Framework (TDF) for Implementing the **My Heart and CKD** Decision Aid.

TDF domain	Supporting quotes from patient and clinician participants
**Capability**	
**Knowledge**	**Patient:** “I think those diagrams will probably help people in that situation that might not be a aware, you know, just that visual thing, I think that’s a good thing to have for sure.” **Clinician:** “we can help them work through it and we can review that specific page and use this tool to supplement my answers as a clinician.” **Clinician**: “I would find this most useful, something that could be printed out, the patient can take with them just as a reminder about a discussion.” **Clinician**: “and I suspect that sometimes this will be kind of managed not by the patient, I can see some patients where the family’s very involved in their care, like elderly patients and some ethnic groups where they try to help the patient with decision-making and I’m sure that in some cases there’s going to need to be a way for family member to help do that.”
**Skills**	**Patient:** “it is, especially if you are using a smartphone or an iPad or something, I find it is difficult. Sometimes I have difficulty doing that.” **Patient:** “I think that maybe older people might want it in paper just because of not being comfortable with computers or tablets” **Clinician:** “Sometimes it’s having the topic discussed at a medical grand rounds, like have a guest speaker that’s knowledgeable in chronic kidney disease and coronary disease and introduce this tool as a major part of that.” **Clinician:** “. . . for me this would be pretty smooth and clear for how I would use that in clinics and it’s not convoluted or oversimplified to the point where it would be annoying for me to use without a patient [present], but I guess this is really meant to be used with the patient together.”
**Memory, attention, and decision processes**	**Clinician:** “I would probably do a version of that. I would enter the data myself, print it out, and then have that discussion, but it wouldn’t be more than like handing the patient a package of information and then coming back. I would be using it more as a discussion tool.” **Clinician:** “then there’s going to be elderly people, but you know, often they have a son or a daughter or somebody who can help them go through it.”
**Motivation**	
**Beliefs about capabilities (confidence)**	**Patient:** “You know it’s always good to know where you are at.” **Clinician:** “it’s nice that you mention there’s the option of printouts on some screens or conclusion. So, I would use that for select patients who like print information also.” **Clinician:** “I would definitely plan to be using it in the current format for, you know, I have this kind of a discussion with the patient, I don’t know, once a month lets say and it would be useful for all of those times or almost all of those times.”
**Social/professional role/identify**	**Clinician:** “I just wonder does it, in terms of efficiency does it need to be a physician that’s guiding? Like I think that I can talk about this tool, like give a preamble, but perhaps even the bedside nurse would be able to help navigate that and then if there’s any questions after rounds, I can come and talk to the patient about it. Because if you are on rounds, sometimes it’s a bit tough to incorporate if you are just waiting for the patient to fill this out and I think that there is probably a bit more value in continuing on with rounds being efficient in terms of time completing those task because I think it’s pretty user friendly and if there’s any questions, maybe a bit of an orientation for the nursing staff that particularly onboard patient to be able to help facilitate that I think would be helpful.” **Clinician:** “yeah so you know, on the cardiology units right, this is just a very common scenario and you could probably arrange for some of the nurses just to have training and understanding of this and to run the tool with the patient that are on the floor with chronic kidney disease who are being considered for angiography.”
**Optimism/pessimism**	**Patient:** “I think that this would be good.” **Clinician:** “I think overall it’s a useful resource for the office or inpatient setting to explain to patient as you use the tool about their options and the risks and benefits of both. So I would find it useful and I definitely would be using this especially in clinic.” **Clinician:** “yeah it would be useful, there’s no question it would be, it’s just about getting the implementation.”
**Opportunity**	
**Environmental context/resources**	***Implementation in an acute care setting*** **Patient:** “I think an iPad would be perfect and again with the summary pages with their history and their summary page. I’ve had patients tell me, I’ve got nothing else to do, I’m trapped here, I can’t even get up to go to the bathroom so I might as well sit and read whatever you gave me. Right, you’ve got a little bit of a captive audience and they are going to be interested because now you’ve told them you are currently having a heart attack. You’ve been given this medication, should you be going to the Cath lab, they are going to read that.”
	**Clinician:** “I foresee this taking time and it is before getting to the Cath lab when this should happen. Because it’s very much a before and after the procedure kind of thing. By the time they get to the Cath lab they’ve already declared that that’s, like they’ve already had the conversation with their treating physicians and they are saying, you know, that they want this procedure to be done.” **Clinician:** “I suspect that it would often take longer using this tool than not using this tool, but for the patient population that is quite fixated on the renal risk then it would be quite helpful.” **Clinician:** “it would be a little bit more challenging in a ward or like in an inpatient setting just with the difficulty getting a computer screen into a patient’s room. It would be more of the summary I would be conveying.” ***Implementation in a clinic setting*** **Clinician:** “the issue again is that I would love to use it. I think that if the patient came with it or had it already done I think I would 100% use it. The issue is that in a busy outpatient clinic practice it is tough to do these things.” **Clinician:** “this could be really helpful for the outpatient population who’s coming in for stable coronary disease because then it can be used in clinic and they can even do the prep work before they come in to see the physician and kind of play through it themselves.” ***Resource needs*** *Patient:* “I think that it would have to be on perhaps maybe a tablet or something that they would give you to use.” **Patient:** “Ok, lets just assume that this thing is going to work, how is a person who is in a hospital bed to do this? Because how do you see a screen and a mouse?” **Clinician:** “so just with infection control and difficulty getting a computer in a patients room, I wouldn’t be doing it with them on the screen, but the output, the infographic of the 100 patients is useful. So, I probably would still aim to use this for select inpatient. I would just be entering the data myself, probably printing out the results and using that as a discussion tool in the patient’s room.” **Clinician:** “You should have somebody else who is doing that background work. This extra work can’t fall on the physician.” *Flexibility of use* **Patient:** “yeah this is going to work for both, like you are going through this with your doctor, but this is also a tool you can use on your own as well, correct?” **Patient:** “this actually might be handy for family members that might be forced to help make these decisions. Like say my husband was next to me and he’s going, I don’t know what to choose.” **Clinician:** “it’s nice that you mention there’s the option to have printouts on some screens or conclusion. So I would use that for select patients who like print information also.” **Clinician:** “especially if they are going to be discussing with their family, having a PDF copy at their disposal to discuss with a family member that might be a nurse or something or whatever. I think having that option is good.”
**Social influences (influenced by others)**	**Clinician:** “so I think that the way to introduce it would be, the easiest group for us to introduce it to would be to the Cath lab doctors and Cath lab doctor could then facilitate introducing it to the pre and post angio nurses. And, then in terms of getting out to the wards that would have to be presented to a section meeting for all of the cardiologists and then that would catch the doctors. I think a cardiology round would be a helpful place to catch that.” **Clinician:** “so this will increase the workload of the nurses for example who help the patient navigate through the app right.” **Clinician:** “I mean as soon as the patient gets some information the better they can think about and so on and so forth, so sooner the better. I’m just saying sometimes even in those non-ST elevation MI or acute coronary syndromes things go very quickly.” **Clinician:** “like set it up for the patient, give it to them, step through and let them do it electronically at their leisure or whatever, I think it would be pretty straightforward to look at it with the patient, go through it with them, answer any questions they have. . .if there was a ward iPad that it could be loaded up and passed to the patient to go through it, I think any physician would be happy to go through it and answer questions that a patient might have. But, I just can’t see them owning the set up part of it, and I think I’m being too critical of my own, I just think there’s always something that is important and physicians would just drop it and walk away and go deal with somebody who’s really sick.”

Both clinicians and patients alike saw benefits in using ***My Heart and CKD***, describing the decision aid as “good” and “useful.” Both groups suggested that it would be helpful to offer the tool in both electronic and paper formats, to accommodate personal preferences and learning styles. The primary barrier identified through interviews was related to time constraints. Whereas patients said they would be happy to take the time to use ***My Heart and CKD***, especially as in-patients, many clinicians expressed concerns about the time required to complete all steps within the decision aid during their time with a patient. Some clinicians said if the patient already had patient specific information collected and filled in, they would be happy to use ***My Heart and CKD*** to go over the results. Some clinicians suggested that nurse practitioners or other care providers, depending on the clinical setting, might be included in the implementation process to help support its use while at the same time ensuring consistent delivery of information to patients by all care providers, although this could add to the workload of nurses.

These findings led us to design the decision aid so that it would support varying use preferences and contexts, encompassing both digital and paper-based forms for use, depending on patient preferences and support available for use of digital technologies. Implementation considerations also influenced the order and arrangement of the content in My Heart and CKD to enhance its integration into the clinical environment of SDM encounters. The decision aid and implementation workflow were developed to be adaptable to variation in the amount of time and discussion that might be required between patients and their care givers or family before assessing patient knowledge and arriving at and documenting a decision.

**Figure 1.**
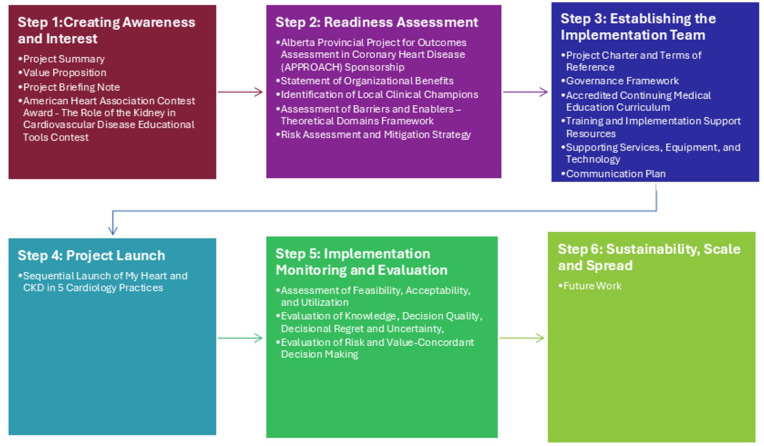
Application of the Can-SOLVE CKD network pathway to implementation for the ***My Heart and CKD*** decision aid.

**Figure 2.**
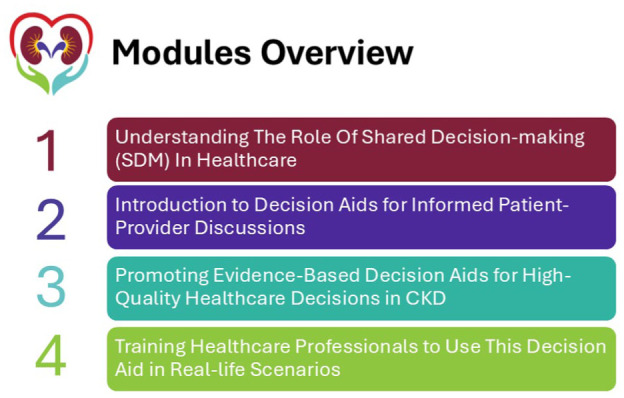
Outline of the training modules created for health care providers to support implementing the My Heart and CKD decision aid.

Findings from our qualitative analyses and pre-clinical simulated patient-physician encounters indicated that additional training and implementation support materials would be needed by users to implement the decision aid. Guided by Can-SOLVE CKD’s 6-Step Pathway to Implementation Guide,^28^ we thus created a collection of documents, resources, education, and training materials to help local teams implement ***My Heart and CKD***. The specific products developed as part of the Implementation Package for ***My Heart and CKD*** and their relationship to each step on the implementation pathway are shown in Figure 1. Select materials are also available in the Supplement. An outline of the accredited CME program is shown in Figure 2.

## Discussion

We have worked across Can-SOLVE CKD’s 4 major pillars, following the six steps within the Pathway to Implementation to develop an implementation strategy for the ***My Heart and CKD*** decision aid. This decision aid is designed to facilitate SDM and support a shift toward more patient-centered care that addresses variability in risks that exist between patients and aligns care with the values and preferences of individual people living with CKD and CAD. Applying HCD brought to light several implementation themes during the decision-aid development process. The TDF allowed us to identify barriers to use as well as potential facilitators, which influenced the design of the decision aid, as well as the development of a suite of implementation training and support materials. This has prepared us to pilot test the decision aid in cardiology practices in Canada and will also support broader implementation scale and spread of the decision aid to additional practices in the future.

Implementation strategies for decision aids need to carefully balance physician expertise while making room for the patient voice in the shared treatment decision-making process. Throughout the project, patients have shaped the design of ***My Heart and CKD*** as well as implementation strategies. Patients have also been involved with selecting the measurements and instruments we will use to measure knowledge, communication, acceptability, and the quality of SDM, to ensure our intervention improves outcomes that matter to patients. Mid-iteration adjustments to the design of the decision aid were made to enhance feasibility of use and comprehension by patients and provide flexible approaches to integration within clinical workflow to increase the probability of implementation success. Patient partners play an important role in knowledge dissemination about ***My Heart and CKD*** to other patients and physicians. As one patient partner advises:

I would say use the tool, go slowly, go carefully, look at all aspects and ask questions. If you don’t understand or you are asking should I, could I, would I? There’s always somebody available to answer any question you have.—**Patient Partner**

Strengths of our project include the insights of patients with lived experience, clinicians with experiences caring for people with CKD and CAD, and application of evidence-informed frameworks, such as HCD, the Ottawa decision support framework, and the TDF in development and implementation of the decision aid. However, we have encountered several limitations. Much of the iterative development and interviews with patients was affected by the COVID-19 pandemic, which required us to use virtual sessions and may have altered people’s preferences and experiences with use of digital health technologies. This could have either enhanced or suppressed identification of certain barriers or facilitators to implementation of a web-based tool in clinical practice. However, ***My Heart and CKD*** is highly flexible for use in different contexts and was designed with these uncertainties in mind. Patients and their health care teams can initiate tool use in either digital or paper-based contexts depending on patient technology comfort levels, ever-evolving use of technology in clinic environments, and workflows. The tool allows easy movement between the two, emphasizing flexibility as a strength to overcome the limitation.

As we are still in early stages of implementation, we do not yet know for certain how our identified barriers and facilitators will translate into real world use. It is uncertain whether the intended flexibility of ways to use the decision aid will translate into implementation success in clinical practice. However, we have created a range of support materials for different audiences and in different modalities, and in-person implementation supports to help each clinical environment determine which form of use work best for their situation. While in-person support may not be possible beyond the pilot trial (ie, a potential limitation of scale and spread planning), we do intend to collect and apply the insights and experiences gleaned from the pilot trial to refine or development resources to support future broader implementation efforts.

## Implications

**Figure 3.**
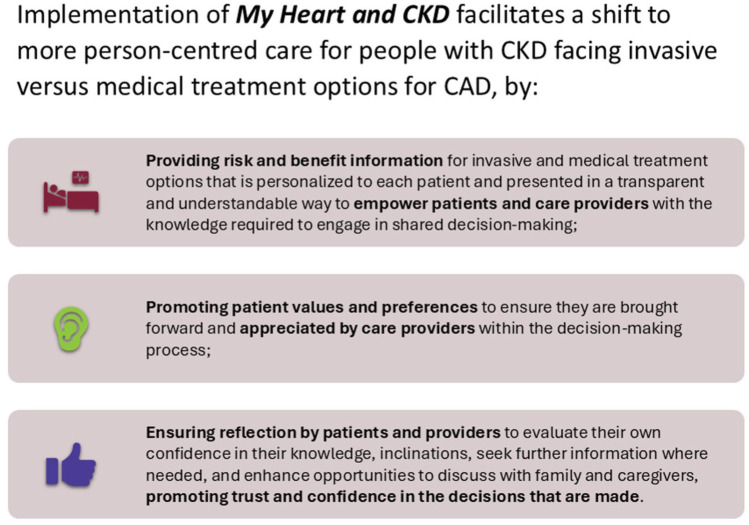
Infographic conveying the value of implementing the My Heart and CKD decision aid.

***My Heart and CKD*** has the potential to be effectively and sustainably implemented within cardiovascular and kidney care, with implications for how care is delivered to people with CAD and CKD. This decision aid promotes personalized, patient-centered care by providing patients with CKD and CAD and their care providers with the tools needed for SDM (Figure 3).

By mobilizing knowledge through decision support, promoting SDM in a manner that addresses known health inequities, and amplifying patient involvement and recognition of their values and preferences in decision-making, this program of work aligns with Can-SOLVE CKD’s mission to transform kidney care and foster equitable health experiences and outcomes for Canadians living with CKD.

## Supplemental Material

sj-pdf-1-cjk-10.1177_20543581251389602 – Supplemental material for My Heart and CKD: Pathway to Implementing a Decision Aid for Patients With CKD and Coronary Artery Disease Supplemental material, sj-pdf-1-cjk-10.1177_20543581251389602 for My Heart and CKD: Pathway to Implementing a Decision Aid for Patients With CKD and Coronary Artery Disease by Julie N. Babione, Pantea Javaheri, Denise Kruger, Todd Wilson, Winnie Pearson, Maureena Loth, Violet March, Wayne Gerber, Bryan J. Har, Michelle M. Graham, Stephen B. Wilton, Krystina B. Lewis and Matthew T. James in Canadian Journal of Kidney Health and Disease
